# Self-Reported Emotions and Facial Expressions on Consumer Acceptability: A Study Using Energy Drinks

**DOI:** 10.3390/foods10020330

**Published:** 2021-02-04

**Authors:** Annu Mehta, Chetan Sharma, Madhuri Kanala, Mishika Thakur, Roland Harrison, Damir Dennis Torrico

**Affiliations:** Department of Wine, Food and Molecular Biosciences, Lincoln University, Lincoln 7647, New Zealand; Annu.Mehta@lincolnuni.ac.nz (A.M.); chetan.sharma@lincoln.ac.nz (C.S.); Madhuri.KanalaManjunath@lincolnuni.ac.nz (M.K.); Mishika.Thakur@lincolnuni.ac.nz (M.T.); Roland.Harrison@lincoln.ac.nz (R.H.)

**Keywords:** emotions, EsSense profile^®^, facial expressions, purchase intention, energy drinks

## Abstract

Emotional responses elicited by foods are of great interest for new product developers and marketing professionals, as consumer acceptance proved to be linked to the emotions generated by the product in the consumers. An emotional measurement is generally considered an appropriate tool to differentiate between the products of similar nutritional value, flavour, liking and packaging. Novel methods used to measure emotions include self-reporting verbal and visual measurements, and facial expression techniques. This study aimed to evaluate the explicit and implicit emotional response elicited during the tasting of two different brands (A and B) of energy drinks. The explicit response of consumers was assessed using liking (nine-point hedonic scale), and emotions (EsSense Profile^®^—Check-All-That-Apply questionnaire), and implicit emotional responses were evaluated by studying facial expressions using the Affectiva Affdex^®^ software. The familiarity of the product and purchase intent were also assessed during the study. The hedonic rating shows a significant difference in liking between the two brands of energy drink during the tasting session. For the explicit emotional responses, participants elicited more positive emotions than the negative emotions for both energy drinks. However, participants expressed “happy”, “active” and “eager” emotions more frequently for energy drink A. On the other hand, the implicit emotional responses through facial expressions indicated a high level of involvement of the participants with energy drink B as compared to energy drink A. The study showed that overall liking and the explicit and implicit emotional measurements are weakly to moderately correlated.

## 1. Introduction

Globalization, which involves a fierce competition among companies to retain consumers and simultaneously to find new ones, is the driving force that keeps food and other industries innovating to make well-informed decisions in the marketplace. In the course of innovation, many concepts are brainstormed, and only a few selective ideas are taken forward for bench- or pilot-scale testing. However, the success of these prototypes in the marketplace is not guaranteed. Indeed, 50–70% of newly launched food products do not last long in the market [1], despite their intensive market research. Sensory and consumer sciences provide a few tools in this context [2,3,4,5] to better understand products [6,7,8,9] and population categories [10], and to minimize the risk of failure [10,11]. One of the most popular and extensively used methods to quantify affective responses in sensory science is the acceptability test using the nine-point hedonic scale [12]. However, product sensory liking does not necessarily always convert into a purchase [13], and formulators should look beyond the liking scores to make a product successful [14]. Thus, many other methods [3,13,15] are being explored to provide insights into food-choice behaviours. A novel sensory and emotional approach is the use of non-verbal cues, such as facial expressions, which can communicate highly detailed information about the individual’s experiences [16] and helps in understanding product liking and different emotions that influence the purchase intention. Emotions play a significant role in the comprehension of food preferences and consumers’ likings. Moreover, emotions are decisive factors in our food choices since the consumed foods can evoke certain emotions [14,17,18]. Several factors affect emotions, such as age, satiety, health, economic condition, and expectations. In addition to these, two types of emotional responses, conscious and unconscious, can be elicited by consumers when exposed to different products [19,20]. However, most market research is based on conscious arousal and measured with self-reporting scales [21,22], such as EsSense Profile^®^ [23] and PrEmo^®^ [24].

Recent studies using the EsSense profile^®^ questionnaire have validated its discriminating power within and between food product categories [25]. The EsSense Profile^®^ questionnaire is cost-effective, easy to use and interpret, covers a wide range of emotions and has provided rich insights into the consumers’ perceptions as well as the liking of products such as beer [26], wine [27,28] and coffee [29,30]. However, the explicit method of emotional measurement requires cognitive thinking to convert the experiences into expressions, which sometimes lose the actual meaning of the emotions felt.

Automatic facial expression recognition (hereafter AFER) is one of the important novel methods to study emotional responses and human behaviours. AFER is a non-verbal and arguably universal language [31] that helps communicate countless emotions, such as happiness, sadness, anger, fear, surprise, and others among humans. Recent studies have been conducted to find the correlation between the AFER and emotions, and how both are comparatively linked with the self-reported likings [32]. Infants and children have been using facial expressions to communicate their emotions and feelings [33], especially in the case of sweet foods, when they smack their lips, protrude the tongue and smile; while, for bitter food, they wrinkle their nose and turn their heads [34]. Researchers have found that facial expressions can recognize and differentiate the basic tastes and odours [35] and found that consumers elicit negative expressions (dislike) more accurately than positive expressions [36]. AFER and autonomous nervous system responses provide insights into food preference in relation to food properties for different kind of foods [37,38,39] and its influence on the purchase behaviours of consumers. The facial expressions can elucidate the consumer acceptability of products based on emotional responses and familiarity. In the present study, energy drinks were selected as the beverage model for evaluating the hedonic and emotional responses of consumers. Energy drinks are soft beverages that contain several stimulants such as caffeine and glucose and have a wide range of flavours and mouthfeels [40], which can affect mood and mental energy [41]. Many studies have proved the effect of caffeine or the combination of caffeine with other ingredients on the mood and cognitive performance [41]. Caffeine in doses of 75 and 150 mg enhances positive emotions (such as happiness and calmness), while it reduces tenseness [42,43] and caffeinated taurine drinks improve alertness [44] and attention [45]. The overall liking and explicit emotion measurements were investigated using a self-reported questionnaire, and implicit emotion measurements were observed from the facial expressions using the Affectiva^®^ software. The study aimed to investigate whether self-reporting liking and explicit emotion measurements provide similar differentiation among the samples. It also evaluated whether the positive/negative emotions elicited during the implicit emotion measurements were correlated to the sensory attributes of the products. The explicit and implicit measurements of emotions on the differentiation of samples were also investigated. Finally, self-reported liking, and explicit and implicit measurements of emotions were studied on the samples’ differentiation. Therefore, the following hypotheses were proposed for this research: H1—The self-reported liking and positive explicit emotion measurement are positively correlated; H2—The positive facial expression emotions and liking will depict similar product differentiation; and H3—The self-reported liking, explicit and implicit emotions, exhibit similar behaviours.

## 2. Material and Methods

### 2.1. Participants

Forty-seven participants (male/female 21/26), who were 20–40 years of age, were recruited via email from Lincoln University for the research experiment. The majority of the participants were students of Asian origin (India (*N* = 27), China (*N* = 13), Vietnam (*N* = 3), Korea (*N* = 1), Hispanic (*N* = 2) and Cambodia (*N* = 1)). The participants were students of Lincoln University and were of Asian origin. Facial expressions vary with different cultures and ethnicities [46,47]; therefore, ethnic-specific facial expressions data were required to understand the implicit emotions depicted by consumers after tasting energy drinks. The participants provided written consent for any sensory deficiency such as anosmia and ageusia, a tasting and video recording session as per ethical requirements—Human Ethics (approval: 2019-68). The selected participants (who self-reportedly did not have any of the previously described sensory deficiencies) were not trained for the experiment, and no prior information regarding the study was disclosed to them. The panellists had previously participated in other focus group studies related to other food products such as chocolates and wines and had experience with this kind of study. The criteria to select panellists were that they should be familiar with the product and consume energy drink at least once a month. The general instructions regarding the procedure were given to the participants, providing information regarding the video recording of their tastings. Participants were asked to look at the camera and focus on evaluating the sensory characteristics of the products. The study was conducted in a sensory laboratory of the Department of Wine, Food and Molecular Biosciences, Lincoln University, New Zealand, which meets the sensory evaluation requirements listed in ISO 6658, 2005 and GB 13868, 2009. In regard to the consumer sample size used, a power analysis to test how this experiment performed was conducted. With a difference in means of 0.81 for overall liking, the power of this experiment was ~0.7; therefore, the probability of Type II error in this experiment is medium to low (~0.3) for this type of consumer’s assessments [48]. In addition, based on an extensive study of acceptability tests, Gacula Jr and Rutenbeck [49] estimated that the correct sample size for the consumer’s evaluations was between 40 and 100 consumers. However, increasing the number of participants can help to minimize the Type II error, increasing the power of the experiment. The samples (~10 mL) were stored and served at a refrigerated temperature of 4 °C in transparent plastic cups marked with three-digit random codes in a white tray. Crackers (Arnotts, Australia) and water were served to rinse the palate after each sample and were asked to have a five-minute break before the next sample to avoid sensory fatigue.

### 2.2. Sample Selection

As a preliminary test, a focused group of four trained panellists evaluated five different brands of energy drinks ((Red Bull, Red Bull GmbH, Salzburg, Austria), (Monster, Monster Beverage Corporation, Corona, CA, USA), (Mother, Monster Beverage Corporation, Corona, CA, USA), (V Guarana, Frucor, Auckland, New Zealand), and (Rockstar, Rockstar, Inc., Las Vegas, NV, USA)) for taste and flavour acceptability. After discussion in a focus group *(N* = 4) and the descriptive analysis of the focus group results, two energy drinks with the highest and the lowest acceptability scores were selected to delineate the difference between the products. These liking differences were used for polarizing the facial emotions that consumers might express during the tasting The Rockstar energy drink manufactured by Rockstar, Inc, The United States (Energy drink A) (*ingredients*: carbonated water, sucrose, glucose, citric acid, taurine, natural and artificial flavours, sodium citrate and caffeine, benzoic acid, caramel colour, sorbic acid, L-cartinine, inositol, niacinamide, calcium pantothenate, milk thistle extract, gingko, biloba leaf extract, guarana seed extract, panax. ginseng root extract, riboflavin, pyridoxine hydrochloride, cyanocobalamin) and V energy drink produced at Frucor, New Zealand (Energy drink B) (*ingredients*: carbonated water, sugar, acidity regulator (citric acid and sodium citrate), taurine, guarana extract (0.12%), colour (caramel), glucuronolactone, caffeine, inositol, vitamins (niacin (B3), pantothenic acid, B6, riboflabin B2, (B12), flavours and contains wheat derivatives) were selected for the experiment. 

### 2.3. Traditional Technique of Acceptance Measurement

The traditional method of acceptability measurement is to ask participants for attributes and overall liking using a 9-point hedonic scale (from 1 = extremely dislike, to 9 = extremely like) [12]. In other words, appearance liking, aroma liking, flavour liking, sweetness liking, bitterness liking, aftertaste liking, mouthfeel liking and overall liking were used for evaluating both energy drinks.

### 2.4. Familiarity and Purchase Intent

The familiarity of the energy drink was assessed using a 5-point scale from 1 (not familiar) to 5 (very familiar). Many studies related to children or adults having proved that in the presence of a familiar person or a product, facial expressions are affected and can cause familiarity bias [50]. Therefore, familiarity was taken as a covariable of the liking experiment in this study. For purchase intent, the binomial scale (Yes or No) was used to answer a question “*Will you purchase this product in the future based on the taste/flavour characteristics?*”

### 2.5. Self-Reported Emotions by EsSense^®^ Profile

A total of 21 emotions from EsSense Profile^®^, such as “active”, “adventurous”, “bored”, “daring”, “disgusting”, “eager”, “energetic”, “good”, “happy”, “interested”, “joyful”, “mild”, “pleasant”, “satisfied”, “warm”, “wild”, “anger”, “sadness”, “surprised”, “fear” and “contempt” were used for this study. The aforementioned emotion terms were selected after consensus by a focus group (*N* = 4). The emotional terms were selected on the basis of the frequency of use (>20%) categorization and in relation to the food tested. The check-all-that-apply (CATA) methodology was used for the consumer study to evaluate the emotions elicited by the energy drinks.

### 2.6. Implicit Emotions by Automated Facial Expression Response Measurement 

**Figure 1:**
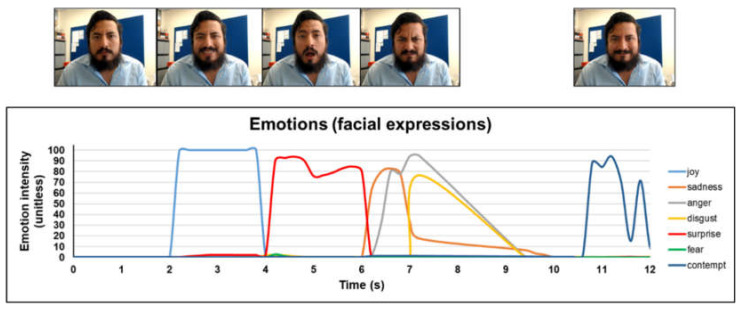
Different emotions: sadness, happy (joy), surprise, disgust, anger, fear, and contempt elicited by a participant. This figure shows 12 s of recording as a demonstration of the different faces’ movements and their relationship with emotions. Actual facial expressions were taken during three seconds after swallowing the sample.

The facial expressions of 30 among 47 panellists were evaluated using Affdex (Affectiva Inc., Waltham, MA, USA) based on the facial inputs. The automated facial coding engine (AFFDEX) was integrated with iMotions Facial Expression Analysis Module (iMotions, Inc., Boston, MA, USA) for decoding the facial emotions using a group of action units (Table 1). The iMotions Facial Expression Analysis Module detects and extracts seven core emotions (joy, anger, fear, disgust, contempt, sadness, and surprise) (shown in Figure 1) and 20 facial expression measures (action units). The action units describe the movements of facial muscles. Emotions are displayed by the movements of a certain number of combined facial muscles. iMotions module also provides timelines annotations, and scores of engagement and valence to provide insight into the facial emotions (https://imotions.com/). The intensity for emotional expression varies from 0 (no expression) to 100 (expression present). The facial expression data collected was from the first three seconds after the participants put the energy drink in their mouths. This is based on previous studies in drinks, in which it was demonstrated that automatic nervous system (AND) responses are expressed immediately after participants are exposed to the stimuli [37,51].

### 2.7. Testing Procedure

The experimental details were explained to participants prior to the commencement of the experiment. The participants were asked to drink 10 mL of the sample in one swallow [52,53], and wait for 15 s, looking straight to the tablet for the recording of the facial expressions. After 15 s, the participants were allowed to fill out the questionnaire section regarding liking and explicit emotions based on a previous study [37]. Therefore, the chances of cognitive bias were minimized. The participants were asked to taste the samples that were served in a 30 mL plastic cups maintained at a temperature of 4 °C and answer the questions related to the sensory attributes, liking, familiarity, purchase intent and emotions felt during the tasting using the questionnaire generated by the RedJade^®^ Sensory Software (RedJade^®^, Martinez, CA, USA). The presentation order of the samples was randomized. The participants tasted the samples under blind conditions; however, facial expressions were recorded for 3 s immediately after the first sip of the sample. The facial expressions of participants were recorded using a camera with a video resolution of 4K (UHD) at 30 FPS (X450, Kaiser Bass, Australia) adjusted in front of participants for the recording of the facial reactions. As a token of appreciation, a can of energy drink was gifted to participants after the experiment.

### 2.8. Statistical Analysis

The hedonic scores of sensory attributes and the liking of the energy drinks during the tasting session were analysed using an analysis of variance (ANOVA). A Tukey test was used as a post hoc data analysis technique to determine the differences between the samples. The significance level (α) was set at 5%. To analyse the relationship between energy drinks and familiarity, the chi-square test for homogeneity was performed. EsSense Profile^®^ data were analysed using the XLSTAT Statistical Software (XLSTAT Version 2019.4.2, Addinsoft, New York, NY, USA). The frequency counts of 21 emotion words which described the samples were calculated. Cochran’s *Q* test was used to find the difference between the samples by evaluating each emotion word used in the CATA questionnaire. The values of purchase intent were statistically analysed for multiple comparisons using the Cochran’s *Q* test. Facial expression data were collected through the Affectiva Affdex (Affectiva Inc., Waltham, MA, USA) software. The analysis of variance (ANOVA) technique, through Minitab^®^ 18 (Version 10.0.17763 Build 17763, State College, PA, USA), was used to locate significant differences among emotions. Pearson correlation coefficients (*r*) among the sensory attributes, familiarity, and facial expressions were calculated and plotted using a correlation matrix. Multivariate analysis of variance (MANOVA) was used to determine the significant difference among facial expressions and correlations among all sensory attributes were tested. Based on the MANOVA results, a principal components analysis (PCA) bi-plot was made. The relationship between each emotion method using overall liking as the response variable was analysed using a linear regression model.

## 3. Results

### 3.1. Traditional Technique of Acceptance Measurement, Familiarity and Purchase Intent

The mean and standard deviation of sensory attributes, overall liking and familiarity of the energy drinks A and B are shown in Table 2. No significant differences (*p* > 0.05) were found between both the energy drinks for all the sensory attributes (appearance, aroma, taste/flavour, sweetness, bitterness, mouthfeel and aftertaste). There was a significant difference in the overall liking of the energy drinks. Energy drink A had a significantly higher overall liking score (6.79) compared to that of energy drink B (5.98). Based on the chi-square test *X2* (4, *N* = 94) = 5.25, *p* = 0.26, no significant difference was found among the samples. The expected value of the last category of the attribute was more than the observed value; therefore, the result’s interpretation was sceptical.

The frequency at which a consumer’s intent to buy energy drinks based on the sensory attributes are shown in Table 3. A total of 68.09% of participants intended to buy energy drink A, while 55.32% of participants expected to buy energy drink B. No significant differences in the purchase intent of energy drink A and B were found.

### 3.2. Self-Reported Emotion Measurements

The frequencies of self-reported emotions data are shown in Table 4. The significant differences in the emotion terms “active” and “interested” were reported in the energy drinks during the tasting. The selection frequency of “active” for energy drink A was 49%, while the selection frequency of “active” for energy drink B was 34%. The selection frequency of “interested” for energy drink A (32%) was almost double than the selection frequency of “interested” for energy drink B (13%). No significant differences were found in other reported emotions for both energy drinks during the tasting. Overall, the participants felt more positive emotions (“good”, “happy”, “interested”, “joyful” and “pleasant”) for energy drink A than that for energy drink B. None of the participants felt “sadness” while tasting sample A or B.

### 3.3. Automated Facial Expression Response Measurements

The mean and standard deviation for the facial expression parameters (joy, sadness, disgust, contempt, anger, fear, surprise, valence, engagement and smile) are shown in Table 5. No significant differences in the facial expressions were reported for both products. Some marginal differences were observed in a few emotion categories, such as “smile”, “engagement”, “joy”, “disgust”, and “surprise”. The intensity of “engagement” was highest for both sample types (2.79 and 1.25 for sample A and B, respectively), followed by “contempt” (2.36 and 0.15 for sample A and B, respectively) and “joy” (0.56 for sample A and 0.97 for sample B). For energy drink B, the participants elicited slightly more “joy” and “smile” compared to that of energy drink A. The intensities of “joy” and “smile” were almost double in energy drink B than that of in energy drink A. The negative emotions such as “sadness”, “anger”, and “fear” were marginally elicited in both sample types.

### 3.4. Correlation and Multivariate Analysis of Hedonic and Facial Expression Responses

**Figure 2:**
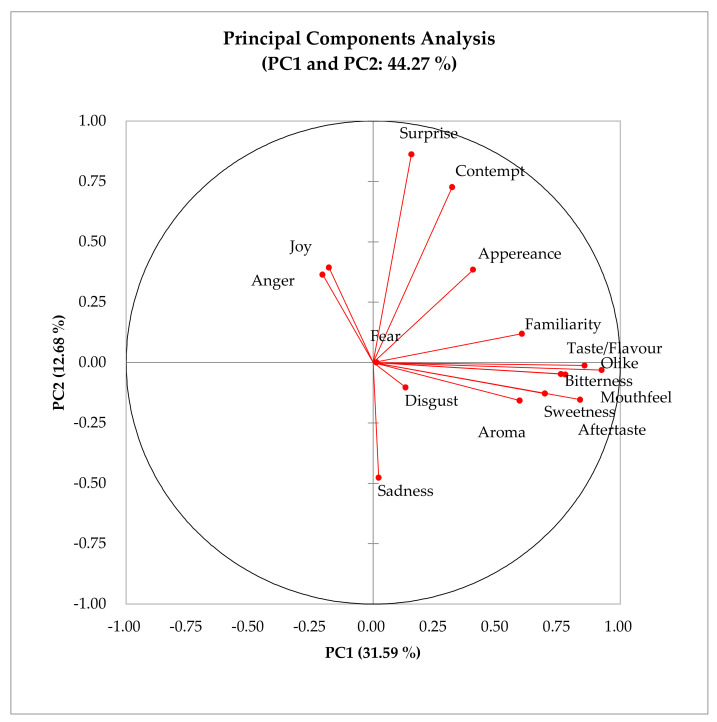
Principal components analysis (PCA) of the sensory attributes, familiarity, liking and emotions of energy drinks.

The Pearson correlation coefficient matrix (Table 6) shows the correlations between various variables (sensory attributes, overall liking, familiarity and AFER) for energy drinks A and B. Familiarity was positively correlated with overall liking (*r* = 0.44) and various sensory attributes such as the liking of taste/flavour (*r* = 0.40), sweetness (*r* = 0.38), bitterness (*r* = 0.44), mouthfeel (*r* = 0.40) and aftertaste (*r* = 0.46). The appearance of the product was negatively correlated to the facial expression of “sadness” (*r* = −0.33). The facial expression “anger” was negatively correlated with the liking of sensory attributes such as sweetness (*r* = −0.38) and aftertaste (*r* = −0.29). The results of the principal components analysis (PCA), which explains the relationship between emotions and hedonic attributes are shown in Figure 2. The principal component one (PC1) explained 31.59% of total inertia, while principal component two (PC2) explained 12.68%, respectively. The overall liking vector was positively related to familiarity, sensory attributes (taste/flavour, mouthfeel, aroma, sweetness, bitterness and aftertaste) and emotions (“disgust” and “sadness”), and negatively correlated to emotions (“joy” and “anger”).

### 3.5. Comparison of Liking with Explicit and Implicit Emotions

The explicit and implicit emotions were examined using a linear regression model with overall liking as a response variable. The difference in regression coefficient and standard error for each explicit and implicit emotions were explained in Table 7. Model 1 shows the significant effect (*p* < 0.05) of explicit emotions on the overall liking of the energy drinks. The explicit emotions “good” and “satisfied” were positively related to overall liking with the regression coefficient values of 1.05 and 0.97, respectively. The explicit emotions “disgusted” and “surprised” were negatively related to overall liking with negative coefficients of −2.17 and −0.82, respectively. The relationship between the implicit emotions and overall liking were explained in Model 2. There was no significant effect (*p* > 0.05) of the emotions from the facial expressions on the overall liking of the samples. The implicit emotions “sadness” and “anger” were negatively associated with overall liking with the linear coefficient values of −23.67 and −43.63, respectively.

## 4. Discussion

### 4.1. Traditional Method, Self-Reported Emotional Measurement and Purchase Intent

In general, energy drink A had higher liking scores than those of energy drink B. The high content of sugar and caffeine present in sample A might have influenced its overall liking. However, no significant differences were found between both samples in the liking of sensory attributes such as appearance, aroma, flavour, sweetness and aftertaste. The familiarity of the product or product category [54] and similar ingredients in both samples can be the reason for not having significant differences in the hedonic ratings of other attributes in the study. Earlier studies showed that the sensory profile of the products made with different ingredients had a strong influence on the hedonic liking scores [55] as compared to products made with similar ingredients.

For the self-elicited emotions, sample A had higher frequency values for positive emotions such as “active”, “good”, “adventurous”, “pleasant”, “joyful”, “contempt”, “warm”, “interested”, and “happy” compared to those of sample B. Sample A had a higher liking score, and also received a higher selection of high-arousal emotion terms such as “adventurous”, and “active”. This result shows that high-arousal emotional terms were important for brand liking in the energy drink category. The selection of high-arousal emotions such as “active” and “adventurous” can be due to the high sugar and caffeine contents in the energy drinks. Specterman et al. (2005) studied that the combined effect of caffeine and glucose had increased excitability and impulsiveness, as blood glucose level increased after consumption [56]. The explicit measurements showed that the positive emotions such as “happy”, “joyful”, and “pleasant” have higher frequency counts as compared to negative emotions such as “sad” and “angry”. These results are in line with earlier findings that consumers use more positive emotions to describe food products than negative emotions [29,57]. In the present study, there was no significant difference in the purchase intent based on the sensory attributes of the energy drinks. Although, the majority of panellists (68%) preferred buying energy drink A rather than energy drink B (55%). This shows that explicit emotions and the overall liking of the product influence the purchase decisions taken by the consumers, as studied earlier [58,59] and plays an essential part in our lives.

### 4.2. Automated Facial Expression Response Measurement

No significant differences in the facial expressions were found. Small sample size could be a reason for the obtained results. Simultaneously, high individual variability in the identified emotions by AFER has previously been reported as an issue in discriminating products [60]. The quasi-absence of the emotions reported through AFER may be attributed to the liquid state of the test samples. Fewer facial movements involved with the liquid state [61,62], absence of apparent emotions [39] evoked by energy drinks or poor emotion recognition by the AFER and higher culture-to-culture (India, China, Cambodia, Vietnam, Korea and Hispanic respondents in this study) or individual-to-individual variances could be, to name a few, some of the reasons for marginal differentiation. Although neutral to positive emotions have been previously found to elicit a few facial expressions [37,63], the efficacy of AFER systems to differentiate between two competing products of a category was not sufficient to replace the existing traditional methods. Pragmatically, products competing in the same category, in general, are not profoundly different from each other, and a high negative valence associated with some of them is not expected. In such cases, the capacity of AFER to differentiate would be limited without the complement of traditional sensory techniques. Culturally specific display rules may also be a reason for the lack of differentiation. These rules govern the amplifying, dampening, or altogether masking of the facial expressions. The use of water, basic taste solutions at varying concentrations and target populations (culturally specific) for calibration or testing may be an option for the efficacy test of AFER systems. A large sample size study using both explicit and implicit methods may shed some light on the topic in the future, but practitioners should complement implicit methods with traditional methods for immediate applications. For future applications, implicit methods can have considerable implications in the retail and foodservice industry, and practitioners should keep testing this methodology.

In comparison to sample A, sample B evoked some negative facial expressions, such as nose scrunch, widen eyes, lip suck, which altogether represent disgust [64] and anger. Contempt and disgust belong to the family of hostile emotions [65]. Limited facial movement hinders the precise measure of some of the facial expressions such as “fear”, “sad” and “anger” [37,63]. Higher attention and engagement observed with sample A may imply that the sample was more enjoyable at the time of tasting. The caffeine, glucose and carbonation in energy drinks enhance the mood and the level of energetic arousal [43] and might be responsible for higher attention and engagement, irrespective of the sample type. More dimpler expressions, though not statistically significant, were observed for sample A. Dimpler has been previously identified as a predictor of positive emotion ratings by machine learning models [66]. In the case of energy drink A, the intensity of dimpler facial movements is higher as compared to energy drink B; thus, exhibiting the higher intensity of emotion contempt during the tasting of energy drink A. The participants elicited a higher intensity of joy and smile in sample B through implicit measures as compared to sample A, which was not the case using the nine-point scale ratings and the explicit study of emotions. Duchenne or genuine smiles are caused by the activation of facial action units 6 and 12 [67], and generally, this is the result of enjoyment and happiness. However, previous studies showed that a smile could be misleading, as many people smile as a sign of embarrassment [68], disappointment [69] or deliberately to hide emotions [70]. This shows that overall liking, explicit emotional responses, and implicit emotional responses vary in the outcome that they have in relationship to hedonic reactions. Explicit methods of emotion measure the conscious and cognitive actions or associations with the food product [14], whereas implicit methods measure the unconscious responses to the stimuli [71]. This finding was in accordance with the study, which stated that the overall liking, self-reported questionnaire and unconscious responses of the consumers are weakly to moderately correlated [37].

### 4.3. Multivariate Analysis of Hedonic and Facial Expression

Based on the Pearson correlation coefficient, familiarity influenced the overall liking of the product significantly. Similar findings were reported in earlier studies of meat [72,73], cheese [74], teas [75] and spirulina-filled pasta [76]. The effect of familiarity on consumers’ preferences of the product also varies with socio-demographic factors [74] as well as cross-culturally [58]. Consumers feel more comfortable with the acquainted brands with which they are satisfied rather than exploring a new one. Familiarity and liking also affect the hedonic ratings of the sensory attributes (taste/flavour, aroma, texture, appearance, sweetness and bitterness) [75], as in the present study, the consumers gave higher liking ratings to the beverage product having familiar sensory attributes. In the case of implicit emotions, the emotion “anger” had been negatively correlated to sweetness and aftertaste. The participants felt less the negative emotion “anger” due to the sweetness and aftertaste of the product. Sweet foods elicited more positive emotions as compared to negative emotions in a previous study [77]. Overall, the results obtained from the self-reported and implicit reactions which provide meaningful insights to understand the differences in energy drinks. Thus, the correlation between emotions and sensory attributes can help food innovators to launch a promising product in a competitive market.

## 5. Conclusions

This preliminary study showed that the samples were significantly different based on overall liking. However, the sensory profiles of the energy drinks were not significantly different due to the similar product category, which also affected the purchase intent of the samples. In the case of explicit emotions, the magnitude of the self-reported positive emotions was slightly higher than negative emotions in both sample types. Higher self-reported positive emotions were reported for energy drink type A compared to B. However, in the case of implicit emotions by automated facial responses, positive emotions were expressed for both energy drinks (A and B). This study concludes that the traditional methods, self-reported emotional measurements and automated facial expression responses can vary in their outcome; however, all these reactions provide meaningful insights into the differentiation of the products. Future studies can be planned to test close- competitors in the same product category with explicit and implicit methods for efficacy-checking of the techniques based on the findings. Non-liquid food categories may produce better differentiation with implicit methods, but a study is needed to validate this assumption. Cultural manifestations, especially those of South and East Asia, in the case of facial expressions, are very subtle and offer another opportunity to test for. The test sensitivity of implicit methods may be more drastically affected with a small sample size; hence, power analysis for implicit methods should be revised. A connection between facial expressions and self-reported emotions could be another important area to study. Finally, based on the experimental findings in this study, it can be concluded that implicit emotion measurement methods are still in their preliminary stages and require many more investigations.

## 6. Limitations

A considerable proportion of the test population was of Asian origin, which would be a limitation in the generalization of these findings over other demographics. Additionally, the limited sample size may pose certain constraints in analysing and interpreting the results. In addition, much recognition is required to understand the effect of the environment on emotions. Therefore, future studies are recommended to employ a greater number of participants and in different contexts such as central location tests and live experience or virtual reality sets to understand the environmental effect on the emotions depicted by facial expression.

## Figures and Tables

**Table 1:** Action units of the facial muscle movements expressing emotions in Affective^®^ https://imotions.com/blog/facial-action-coding-system/.

Emotion	Action Units	Description
Happiness/joy	6 + 12	Cheek raiser, lip corner puller
Sadness	1 + 4 + 15	Inner brow raiser, brow lowerer, lip corner depressor
Surprise	1 + 2 + 5 + 26	Inner brow raiser, outer brow raiser, upper lid raiser, jaw drop
Fear	1 + 2 +4 + 5 + 7 + 20 + 26	Inner brow raiser, outer brow raiser, brow lowerer, upper lid raiser, lid tightener, lip stretcher, jaw drop
Anger	4 + 5 + 7 + 23	Brow lowerer, upper lid raiser, lid tightener, lip tightener
Disgust	9 + 15 + 16	Nose wrinkler, lip corner depressor, lower lip depressor
Contempt	12 + 14 (on one side of the face)	Lip corner puller, dimpler

**Table 2:** Sensory attributes, overall liking and familiarity of energy drink A and B after tasting.

Attributes	Energy Drink A	Energy Drink B	F Value *	Pr > F *
Appearance	6.96 ± 1.33 ^a^	6.53 ± 1.38 ^a^	2.31	0.13
Aroma	6.89 ± 1.70 ^a^	6.40 ± 1.51 ^a^	2.18	0.14
Flavour	6.72 ± 1.75 ^a^	6.02 ± 1.94 ^a^	3.39	0.07
Sweetness	6.55 ± 1.82 ^a^	6.23 ± 1.90 ^a^	0.69	0.41
Bitterness	5.75 ± 1.93 ^a^	5.32 ± 1.82 ^a^	1.21	0.27
Mouthfeel	6.75 ± 1.91 ^a^	6.15 ± 2.03 ^a^	2.15	0.15
Aftertaste	6.28 ± 2.03 ^a^	5.51 ± 2.17 ^a^	3.13	0.08
Overall liking	***6.79 ± 1.67 ^a^***	***5.98 ± 2.03 ^b^***	***4.46***	***0.04***
Familiarity **	2.72 ± 1.30 ^a^	2.43 ± 1.02 ^a^	*X2* = 5.25	*p* = 0.26

Sensory attributes and overall liking of energy drink A and energy drink B were measured by 9-point hedonic scale (1 = extremely disliked and 9 = extremely liked) and familiarity between the energy drinks were assessed with 5-point categorical scale (1 = not at all familiar and 5 = extremely familiar). Bold italicized values indicate that the parameter was significantly different from 0 (*p* < 0.05). * F value = mean square or mean square error. Effects were considered significant if Pr (probability) > F was <0.05 (bold probability and F value). ** Familiarity was analysed by chi-square at a 95% confidence level. ^a,b^ Means with different superscripts in each row indicate significant differences (*p* < 0.05).

**Table 3:** Purchase intent frequencies of energy drinks during the tasting session.

Energy Drinks	Willingness to Purchase (%)
A	68.09 ^a^
B	55.32 ^a^

Cochran’s *Q* is used to find the difference between energy drinks. The table shows a percentage of consumers willing to buy the energy drink after the tasting session of the energy drinks. Different superscript letters in each row indicate significant differences (*p* < 0.05).

**Table 4:** Emotions felt after tasting energy drink A and B from Cochran’s *Q* test.

Attributes	Energy Drink A	Energy Drink B
Active	*****0.49 ^a^*****	*****0.34 ^b^*****
Adventurous	0.19 ^a^	0.13 ^a^
Bored	0.11 ^a^	0.04 ^a^
Daring	0.06 ^a^	0.06 ^a^
Disgusted	0.06 ^a^	0.11 ^a^
Eager	0.11 ^a^	0.13 ^a^
Energetic	0.36 ^a^	0.43 ^a^
Good	0.49 ^a^	0.40 ^a^
Happy	0.28 ^a^	0.15 ^a^
Interested	*****0.32 ^a^*****	*****0.13 ^b^*****
Joyful	0.30 ^a^	0.15 ^a^
Mild	0.26 ^a^	0.19 ^a^
Pleasant	0.34 ^a^	0.28 ^a^
Satisfied	0.26 ^a^	0.26 ^a^
Warm	0.15 ^a^	0.09 ^a^
Wild	0.09 ^a^	0.09 ^a^
Anger	0.02 ^a^	0.02 ^a^
Sadness	0.00 ^a^	0.00 ^a^
Surprised	0.11 ^a^	0.23 ^a^
Fear	0.04 ^a^	0.02 ^a^
Contempt	0.11 ^a^	0.09 ^a^

A check-all-that-apply (CATA) questionnaire was used to select emotions related to the sample, and Cochran’s *Q* is used to find the difference between the products. Bold italicized values indicate that the parameter was significantly different (*p* < 0.05). ^a,b^ Means with different superscripts in each row indicate significant differences (*p* < 0.05).

**Table 5:** Facial expressions and facial features during the tasting of energy drinks A and B.

Parameters	Energy Drink A	Energy Drink B	F Value	Pr > F *
Engagement	2.79 ± 8.27 ^a^	1.25 ± 5.11 ^a^	0.54	0.47
Contempt	2.36 ± 10.07 ^a^	0.15 ± 0.07 ^a^	1.25	0.27
Smile	0.83 ± 3.07 ^a^	1.24 ± 5.11 ^a^	0.1	0.75
Joy	0.56 ± 2.55 ^a^	0.97 ± 4.87 ^a^	0.12	0.73
Valence	0.44 ± 2.51 ^a^	0.52 ± 4.63 ^a^	0.01	0.95
Disgust	0.36 ± 0.22 ^a^	0.78 ± 2.10 ^a^	0.86	0.36
Surprise	0.30 ± 0.43 ^a^	0.17 ± 0.08 ^a^	2.44	0.13
Sadness	0.02 ± 0.01 ^a^	0.02 ± 0.01 ^a^	0.001	0.98
Anger	0.02 ± 0.00 ^a^	0.02 ± 0.00 ^a^	0.16	0.69
Fear	0.00 ± 0.00 ^a^	0.01 ± 0.015 ^a^	1.13	0.29
Attention	67.90 ± 29.15 ^a^	62.49 ± 30.10 ^a^	0.39	0.54
Eye closure	28.12 ± 41.23 ^a^	18.96 ± 35.57 ^a^	0.67	0.42
Mouth open	2.43 ± 10.18 ^a^	0.58 ± 1.39 ^a^	0.84	0.36
Dimpler	2.38 ± 7.72 ^a^	0.15 ± 0.44 ^a^	2.19	0.15
Smirk	2.12 ± 9.00 ^a^	0.13 ± 0.54 ^a^	1.27	0.27
Brow raise	2.03 ± 6.33 ^a^	0.36 ± 0.85 ^a^	1.79	0.19
Lip suck	1.81 ± 5.44 ^a^	1.91 ± 8.75 ^a^	0.00	0.97
Inner brow raises	0.72 ± 1.86 ^a^	0.71 ± 2.76 ^a^	0.00	0.99
Lip press	0.41 ± 1.20 ^a^	0.08 ± 0.16 ^a^	1.88	0.18
Jaw drop	0.40 ± 0.68 ^a^	0.21 ± 0.25 ^a^	1.77	0.19
Chin raise	0.27 ± 0.69 ^a^	0.93 ± 3.79 ^a^	0.63	0.43
Lid tighten	0.23 ± 0.47 ^a^	0.31 ± 0.69 ^a^	0.17	0.68
Nose wrinkle	0.16 ± 0.41 ^a^	2.24 ± 7.55 ^a^	1.59	0.21
Brow furrow	0.09 ± 0.30 ^a^	0.21 ± 0.79 ^a^	0.38	0.54
Lip pucker	0.08 ± 0.17 ^a^	0.09 ± 0.18 ^a^	0.03	0.87
Cheek raise	0.04 ± 0.14 ^a^	0.02 ± 0.04 ^a^	0.26	0.61
Lip stretch	0.04 ± 0.14 ^a^	1.11 ± 5.63 ^a^	0.76	0.39
Eye widen	0.02 ± 0.04 ^a^	1.36 ± 5.35 ^a^	1.31	0.26
Upper lip raise	0.02 ± 0.07 ^a^	0.31 ± 1.06 ^a^	1.63	0.21
Lip corner depressor	0.01 ± 0.02 ^a^	0.01 ± 0.02 ^a^	0.03	0.86

Means values of the different facial expressions with different superscripts in each row indicate a significant difference (*p* < 0.05). * F value = Mean square or mean square error. Effects were considered significant if Pr (probability) > F was <0.05 (bold probability and F value). ^a^ Means with different superscripts in each row indicate significant differences (*p* < 0.05).

**Table 6:** Pearson correlation coefficient matrix among the sensory attributes, familiarity, liking and emotions for energy drinks.

Variables	Appearance	Aroma	Flavour	Sweetness	Bitterness	Mouthfeel	Aftertaste	Olike	Familiarity	Joy	Sadness	Disgust	Contempt	Anger	Fear	Surprise
Appearance	**1.00**	0.21	**0.39**	**0.33**	0.14	**0.32**	0.15	**0.36**	0.20	0.10	**−0.33**	0.14	0.11	0.18	0.01	0.20
Aroma	0.21	**1.00**	**0.56**	**0.32**	**0.38**	**0.34**	**0.39**	**0.53**	0.28	−0.14	0.02	−0.01	0.12	−0.11	0.03	−0.12
Flavour	**0.39**	**0.56**	**1.00**	**0.67**	**0.56**	**0.50**	**0.65**	**0.81**	**0.40**	−0.06	0.15	0.21	0.20	−0.11	−0.01	0.18
Sweetness	**0.33**	**0.32**	**0.67**	**1.00**	**0.38**	0.26	**0.59**	**0.57**	**0.38**	−0.19	−0.02	0.14	0.13	**−0.38**	0.03	0.02
Bitterness	0.14	**0.38**	**0.56**	**0.38**	**1.00**	**0.70**	**0.67**	**0.72**	**0.44**	−0.12	0.10	0.09	0.19	0.05	−0.03	0.08
Mouthfeel	**0.32**	**0.34**	**0.50**	0.26	**0.70**	**1.00**	**0.62**	**0.75**	**0.40**	−0.12	0.16	0.10	0.21	−0.11	−0.08	0.06
Aftertaste	0.15	**0.39**	**0.65**	**0.59**	**0.67**	**0.62**	**1.00**	**0.80**	**0.46**	−0.09	−0.07	0.11	0.15	**−0.29**	0.00	−0.03
Olike	**0.36**	**0.53**	**0.81**	**0.57**	**0.72**	**0.75**	**0.80**	**1.00**	**0.44**	−0.10	0.01	0.11	0.21	−0.10	0.06	0.09
Familiarity	0.20	0.28	**0.40**	**0.38**	**0.44**	**0.40**	**0.46**	**0.44**	**1.00**	−0.21	−0.07	−0.21	0.27	−0.14	0.13	0.18
Joy	0.10	−0.14	−0.06	−0.19	−0.12	−0.12	−0.09	−0.10	−0.21	**1.00**	−0.27	−0.02	−0.03	0.13	−0.07	0.18
Sadness	**−0.33**	0.02	0.15	−0.02	0.10	0.16	−0.07	0.01	−0.07	−0.27	**1.00**	0.13	−0.22	0.00	0.02	−0.08
Disgust	0.14	−0.01	0.21	0.14	0.09	0.10	0.11	0.11	−0.21	−0.02	0.13	**1.00**	−0.05	0.01	0.02	0.02
Contempt	0.11	0.12	0.20	0.13	0.19	0.21	0.15	0.21	0.27	−0.03	−0.22	−0.05	**1.00**	−0.08	−0.06	**0.80**
Anger	0.18	−0.11	−0.11	**−0.38**	0.05	−0.11	**−0.29**	−0.10	−0.14	0.13	0.00	0.01	−0.08	**1.00**	0.11	0.24
Fear	0.01	0.03	−0.01	0.03	−0.03	−0.08	0.00	0.06	0.13	−0.07	0.02	0.02	−0.06	0.11	**1.00**	0.04
Surprise	0.20	−0.12	0.18	0.02	0.08	0.06	−0.03	0.09	0.18	0.18	−0.08	0.02	**0.80**	0.24	0.04	**1.00**

Bold values show significant (*p* < 0.05) positive and negative correlations.

**Table 7:** A linear regression model * for each emotion method using overall liking as the response variable.

Model **	Emotion Parameter	Regression Coefficient	Standard Error (SE)	*p* Value	Regression Parameters		R^2^
					Overall	MSE	
					Mean		
**Model 1 (self-reported emotions from CATA)**	Active	0.51	0.34	0.14	5.75	1.87	0.59
	Adventurous	0.11	0.46	0.81			
	Bored	−0.15	0.63	0.81			
	Daring	0.87	0.74	0.24			
	***Disgusted***	*>**−2.17***	*>**0.58***	*>**<0.01***			
	Eager	−0.61	0.50	0.23			
	Energetic	−0.11	0.35	0.76			
	*>**Good***	*>**1.05***	*>**0.32***	*>**<0.01***			
	Happy	0.59	0.40	0.15			
	Interested	0.39	0.40	0.34			
	Joyful	0.02	0.38	0.96			
	Mild	−0.36	0.41	0.38			
	Pleasant	0.27	0.35	0.44			
	*>**Satisfied***	*>**0.97***	*>**0.35***	*>**0.01***			
	Warm	0.15	0.50	0.76			
	Wild	−0.99	0.64	0.13			
	Anger	−0.14	1.21	0.91			
	*>**Surprised***	*>**−0.82***	*>**0.40***	*>**0.04***			
	Fear	−1.14	0.94	0.23			
	Contempt	−0.28	0.52	0.59			
**Model 2 (facial expressions)**	Joy	4.66	7.71	0.55	5.66	4.06	0.05
	Sadness	−23.67	52.73	0.66			
	Disgust	0.13	0.19	0.51			
	Contempt	8.95	10.72	0.41			
	Anger	−43.63	112.84	0.70			
	Fear	7.47	38.04	0.85			
	Surprised	−1.98	11.85	0.87			

* Data points were fitted using a linear regression model with overall liking as the response variable. MSE represents the mean square error value. R^2^ is the coefficient of determination of the regression models. ** Bold italicized values indicate that the parameter was significantly different from 0 (*p* < 0.05).

## Data Availability

The data presented in this study are available on request from the corresponding author.
